# Gamma Interferon Is Required for *Chlamydia* Clearance but Is Dispensable for T Cell Homing to the Genital Tract

**DOI:** 10.1128/mBio.00191-20

**Published:** 2020-03-17

**Authors:** Jennifer D. Helble, Rodrigo J. Gonzalez, Ulrich H. von Andrian, Michael N. Starnbach

**Affiliations:** a Department of Microbiology, Harvard Medical School, Boston, Massachusetts, USA; b Department of Immunology, Harvard Medical School, Boston, Massachusetts, USA; c Ragon Institute of MGH, MIT and Harvard, Cambridge, Massachusetts, USA; University of Oklahoma Health Sciences Center

**Keywords:** *Chlamydia*, IFN-γ, T cells, genital tract immunity, interferons, mucosal immunity, mucosal pathogens

## Abstract

While there is no effective vaccine against Chlamydia trachomatis infection, previous work has demonstrated the importance of C. trachomatis-specific CD4^+^ T cells (NR1 T cells) in pathogen clearance. Specifically, NR1 T cells have been shown to be protective in mice, and this protection depends on the host’s ability to sense the cytokine gamma interferon (IFN-γ). However, it is unclear what role NR1 production or sensing of IFN-γ plays in T cell homing to the genital tract or T cell-mediated protection against C. trachomatis. Using two-photon microscopy and flow cytometry, we found that naive wild-type (WT), IFN-γ^−/−^, and IFN-γR^−/−^ NR1 T cells specifically home to sections in the genital tract that contain C. trachomatis. We also determined that protection against infection requires production of IFN-γ from either NR1 T cells or endogenous cells, further highlighting the importance of IFN-γ in clearing C. trachomatis infection.

## Abstract (precis)

Chlamydia trachomatis is an important mucosal pathogen that is the leading cause of sexually transmitted bacterial infections in the United States. Despite this, there is no vaccine currently available. In order to develop such a vaccine, it is necessary to understand the components of the immune response that can lead to protection against this pathogen. It is well known that antigen-specific CD4^+^ T cells are critical for *Chlamydia* clearance, but the contexts in which they are protective or not protective are unknown. Here, we aimed to characterize the importance of gamma interferon production and sensing by T cells and the effects on the immune response to C. trachomatis. Our work here helps to define the contexts in which antigen-specific T cells can be protective, which is critical to our ability to design an effective and protective vaccine against C. trachomatis.

## INTRODUCTION

The human pathogen Chlamydia trachomatis is the most commonly reported sexually transmitted infection in the United States, with an estimated 3 million cases per year (1, 2). While typically treated with antibiotics, untreated genital infections can lead to downstream diseases, including pelvic inflammatory disease, ectopic pregnancy, and infertility (3). Ultimately, the best way to treat this epidemic is through the development of a vaccine. Recent vaccine efforts have highlighted the importance of mucosal priming in the generation of protection against C. trachomatis (4, 5). Mucosal priming is crucial for generating a protective antigen-specific CD4^+^ T cell population that can establish tissue residency in the genital tract, allowing rapid clearance of the pathogen upon challenge (4). In developing a *Chlamydia* vaccine that elicits an effective CD4^+^ T cell response, it is critical to thoroughly understand the precise circumstances under which CD4^+^ T cells are protective.

*Chlamydia*-specific T cell receptor transgenic T cells represent a valuable tool to address questions regarding antigen-specific T cells in naive mice (6–8). We have previously established one such transgenic mouse, denoted the NR1 mouse, in which CD4^+^ T cells are specific for the C. trachomatis protein Cta1 (6). Following C. trachomatis infection, NR1 T cells can home to the genital tract using specific chemokine receptors (9) and host integrins (10) that are similar to those used by endogenous T cells (11–13). NR1 T cell homing to the genital tract is essential for C. trachomatis clearance, as T cells that cannot home to the genital tract are unable to clear infection (9, 10).

NR1 T cells have also been found to be protective in mice both when skewed to a Th1 phenotype (14, 15) or during secondary infection (4). Th1 T cells are typically characterized by their production of the cytokine gamma interferon (IFN-γ). Indeed, it has been shown that IFN-γ is essential for host clearance of *Chlamydia* (14–19). It is thought that antigen-specific CD4^+^ T cells can help control infection through their production of IFN-γ, as endogenous C. trachomatis-specific CD4^+^ T cells (14) and transgenic CD4^+^ T cells corresponding to both C. trachomatis and the mouse-adapted pathogen C. muridarum (8, 14, 15) have all been shown to produce IFN-γ. However, it is unknown if antigen-specific T cell production or sensing of IFN-γ is absolutely required for homing to the genital tract or for clearing infection.

In this study, we sought to determine the role of IFN-γ production and sensing by C. trachomatis-specific CD4^+^ T cells in the context of C. trachomatis genital tract infection. To this end, we generated NR1 T cells that were deficient in IFN-γ production (IFN-γ^−/−^ cells) or in IFN-γ sensing (IFN-γR^−/−^ cells). We found that IFN-γ production and sensing are not required for T cell homing to the genital tract tissue as a whole or for homing to specific sites within the genital tract that contain bacteria. However, in the absence of host IFN-γ production, IFN-γ production but not sensing by NR1 T cells is required to clear C. trachomatis infection. Our data suggest that IFN-γ plays a key role as an effector cytokine in clearing C. trachomatis infection but does not mediate T cell homing.

## RESULTS

### NR1 T cells deficient in IFN-γ production or sensing are equally effective at homing to the genital tract following Chlamydia trachomatis infection.

**FIG 1:**
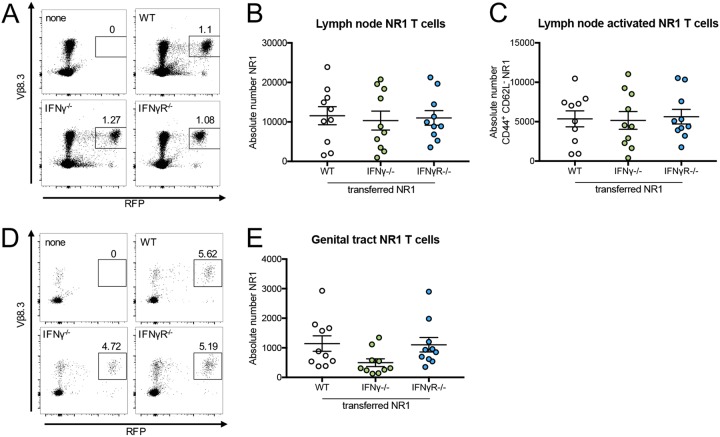
NR1 T cells deficient in IFN-γ production or sensing are equally effective at homing to the genital tract following Chlamydia trachomatis infection. B6 mice received 10^6^ wild-type (WT), IFN-γ^−/−^, or IFN-γR^−/−^ RFP NR1 T cells 1 day prior to infection with 5 × 10^6^ IFU C. trachomatis. (A to C) Five days postinfection, uterine draining lymph nodes were assessed by flow cytometry for (A and B) NR1 T cells (RFP^+^ Vβ8.3^+^ of CD4^+^) and (C) activated NR1 T cells (CD44^+^ CD62L^−^ of NR1^+^). (D and E) Upper genital tracts were assessed by flow cytometry for NR1 T cells. Data were pooled from results of two independent experiments performed with at least five mice per group and were analyzed using an ordinary one-way analysis of variance (ANOVA) with Dunnett’s multiple-comparison test.

Antigen-specific CD4^+^ T cells from T cell receptor transgenic NR1 mice showing specificity to C. trachomatis use certain chemokine receptors and host integrins to traffic to the genital tract (9, 10); however, it is unclear whether or not IFN-γ production or sensing by these cells also plays a role. To address this issue, we generated NR1 mice that were deficient in IFN-γ production (IFN-γ^−/−^ mice) or IFN-γ sensing (IFN-γR^−/−^ mice) and transferred the relevant cells into wild-type (WT) B6 mice. One day after transfer, mice were inoculated transcervically with C. trachomatis (14). Five days postinoculation, the upper genital tract and draining iliac lymph nodes were harvested and NR1 T cell populations were assessed by flow cytometry using red fluorescent protein-positive (RFP^+^) Vβ8.3^+^ gating. Both IFN-γ^−/−^ and IFN-γR^−/−^ NR1s trafficked to the draining lymph nodes (Fig. 1A and B) and were activated (CD44^+^ CD62L^−^) similarly to transferred WT control cells (Fig. 1C). We also found that trafficking to the draining iliac lymph nodes was dependent on the presence of infection, as NR1 T cells were unable to home to iliac lymph nodes in uninfected mice or to nondraining lymph nodes in infected mice (see Fig. S1 in the supplemental material). All three types of NR1 T cells were also able to home to the genital tract (Fig. 1D and E) and did so to similar extents. These data indicate that IFN-γ production and sensing are not required for naive NR1 T cell homing to the genital tract following C. trachomatis infection and that they likely use alternative chemokine receptors (9, 10) to traffic to the site of infection.

10.1128/mBio.00191-20.1 FIG S1 NR1 T cells traffic to the draining iliac lymph nodes in an antigen-dependent manner that is independent of IFN-γ production or sensing. WT B6 mice received 10^6^ WT, IFN-γ^−/−^, or IFN-γR^−/−^ RFP NR1 T cells 1 day prior to infection with 5 × 10^6^ IFU C. trachomatis or inoculation with PBS. Five days postinfection, draining iliac lymph nodes and nondraining brachial lymph nodes were assessed by flow cytometry for (A and B) NR1 T cells (RFP^+^ Vβ8.3^+^ of CD4^+^) and (C) activated NR1 T cells (CD44^+^ CD62L^−^ of NR1^+^). Data are representative of results from one experiment performed with five mice per group and were analyzed using a two-way analysis of variance (ANOVA) with Dunnett’s multiple-comparison test. ****, *P < *0.0001. Download FIG S1, PDF file, 0.1 MB. Copyright © 2020 Helble et al. 2020 Helble et al. This content is distributed under the terms of the Creative Commons Attribution 4.0 International license.

### NR1 T cells localize to sections of the genital tract containing C. trachomatis.

**FIG 2:**
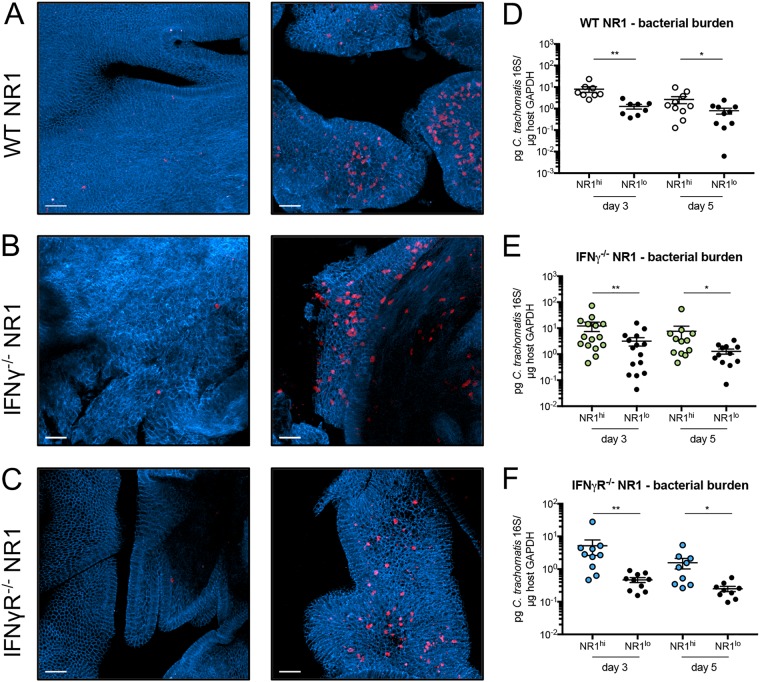
NR1 T cells localize to sections of the genital tract containing C. trachomatis. E-cadherin–CFP mice (A to C) or B6 mice (D to F) received 10^6^ (A and D) WT RFP NR1 T cells, (B and E) IFN-γ^−/−^ RFP NR1 T cells, or (C and F) IFN-γR^−/−^ RFP NR1 T cells 1 day prior to transcervical infection with 5 × 10^6^ IFU C. trachomatis. (A to C) Five days postinfection, upper genital tracts were harvested and visualized by two-photon microscopy for NR1 T cell homing. Images are maximum projections obtained from a depth up to 100 μm, and scale bars denote 50 μm. (D to F) At 3 and 5 days postinfection, genital tracts were subdivided into sections visibly containing NR1 T cells (NR1^hi^) or not containing NR1 T cells (NR1^lo^). Tissue sections were analyzed by quantitative PCR (qPCR) for bacterial burden. Data shown in panels D to F were pooled from results of two independent experiments performed with at least five mice each and were analyzed using Wilcoxon matched-pairs signed rank test. *, *P < *0.05; **, *P < *0.01.

**FIG 3:**
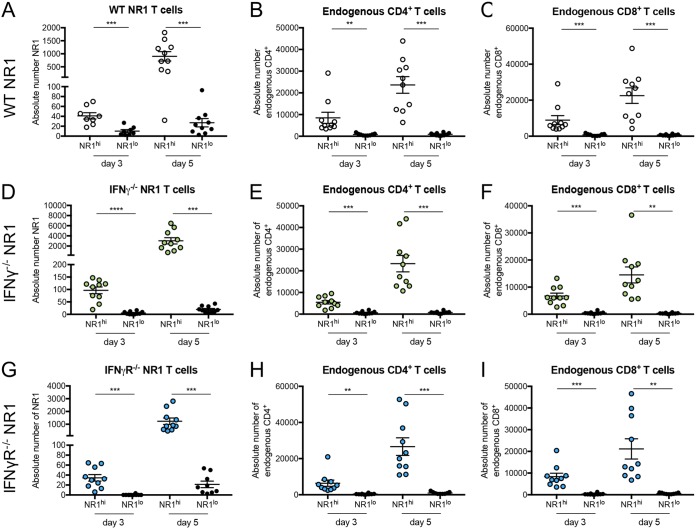
Endogenous CD4^+^ and CD8^+^ T cells also localize to sections in the genital tract containing NR1 T cells. B6 mice received 10^6^ (A to C) WT RFP NR1, (D to F) IFN-γ^−/−^ RFP NR1, or (G to I) IFN-γR^−/−^ RFP NR1 T cells 1 day prior to transcervical infection with 5 × 10^6^ IFU C. trachomatis. At 3 and 5 days postinfection, genital tracts were subdivided into sections visibly containing NR1 T cells (NR1^hi^) or not containing NR1 T cells (NR1^lo^) and assessed by flow cytometry for the number of (A, D, and G) NR1 T cells, (B, E, and H) endogenous CD4^+^ T cells (CD4^+^ CD8^−^ of CD3^+^), and (C, F, and I) endogenous CD8^+^ T cells (CD8^+^ CD4^−^ of CD3^+^). Data were pooled from at least two independent experiments performed with five mice each and were analyzed using paired *t* tests. **, *P < *0.01; ***, *P < *0.001; ****, *P < *0.0001.

Given that there was no difference in T cell homing in general, we next sought to look more directly at the upper genital tract to address any changes in spatial distribution between WT, IFN-γ^−/−^, and IFN-γR^−/−^ NR1 T cells during infection. To do this, we used two-photon microscopy, which allows us to visualize the entire uterine mucosa without the need for sectioning. We transferred WT, IFN-γ^−/−^, or IFN-γR^−/−^ NR1 T cells to E-cadherin–CFP (E-cadherin–cyan fluorescent protein) mice and inoculated the following day. Five days postinoculation, upper genital tracts were harvested, fixed, and analyzed through two-photon microscopy. Interestingly, we found that the T cells were distributed unequally throughout the tissue. Some sites within the genital tract contained very few NR1 T cells, regardless of genotype (Fig. 2A to C, left), while other areas contained a substantial number of NR1 T cells (Fig. 2A to C, right). Given the antigen-specific nature of the NR1 T cells, we hypothesized that they localized to specific sections in the tissue that contained C. trachomatis. To test this, we harvested the genital tract from B6 mice at 3 and 5 days postinoculation and subdivided the uterine horns into segments visibly containing NR1 T cells (NR1^hi^) and visibly lacking NR1 T cells (NR1^lo^) with the aid of a fluorescence dissecting microscope and confirmed the results by flow cytometry (Fig. 3A, D, and G). We found that WT, IFN-γ^−/−^, and IFN-γR^−/−^ NR1 T cells all homed to specific sites in the genital tract that contained a higher bacterial burden (Fig. 2D to F). NR1^hi^ segments also contained greater numbers of endogenous CD4^+^ and CD8^+^ T cells (Fig. 3B, C, E, F, H, and I). Together, our data suggest that both antigen-specific and endogenous T cells can localize to the specific sites within the genital tissue containing bacteria and that IFN-γ plays a minimal role in directing NR1 T cell localization to specific sites within the genital tract that contain C. trachomatis.

### IFN-γ production by NR1 T cells is necessary for NR1-mediated protection against C. trachomatis.

**FIG 4:**
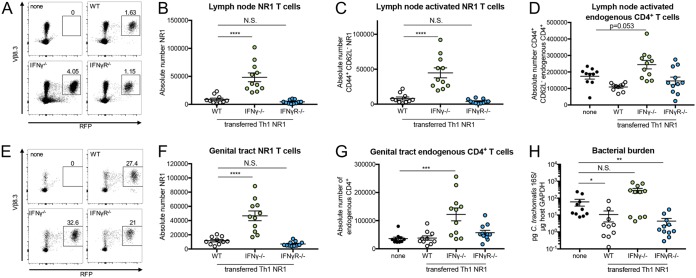
IFN-γ production by NR1 T cells is necessary for NR1-mediated protection against C. trachomatis. IFN-γ^−/−^ mice received 10^6^ Th1-skewed WT, IFN-γ^−/−^, or IFN-γR^−/−^ RFP NR1 T cells 1 day prior to infection with 5 × 10^6^ IFU C. trachomatis. (A to D) Five days postinfection, uterine draining lymph nodes were assessed by flow cytometry for (A and B) NR1 T cells, (C) activated NR1 T cells, and (D) activated endogenous CD4^+^ T cells (CD44^+^ CD62L^−^ of CD4^+^ RFP^−^). (E to H) Upper genital tracts were assessed by flow cytometry for (E and F) NR1 T cells and (G) endogenous CD4^+^ T cells and by qPCR for (H) bacterial burden. Data were pooled from results of two independent experiments performed with at least five mice per group and were analyzed by ordinary one-way ANOVA with Dunnett’s multiple-comparison test (B to D, F, and G) or by Kruskal-Wallis test with Dunn’s multiple-comparison test (H). *, *P < *0.05; **, *P < *0.01; ***, *P < *0.001; ****, *P < *0.0001; N.S., not significant.

It is known that host IFN-γ sensing during *Chlamydia* infection is necessary for bacterial clearance (15) and that T cell-mediated clearance of *Chlamydia* relies on Th1 T cells, which are typically characterized by IFN-γ production (15, 16). Indeed, it has previously been established that NR1 T cells skewed to a Th1 phenotype can protect mice that are deficient in IFN-γ (14, 15). Thus, we were curious if antigen-specific NR1 T cells that were defective for IFN-γ production or IFN-γ sensing would provide a similar level of protection. To address this issue, WT, IFN-γ^−/−^, and IFN-γR^−/−^ NR1 T cells were skewed to a Th1 phenotype *in vitro* prior to transfer into IFN-γ^−/−^ mice (Fig. S2). The WT and IFN-γ^−/−^ NR1 T cells were skewed efficiently to a T-bet^+^ phenotype. However, the IFN-γR^−/−^ NR1 T cells were skewed slightly less efficiently, which likely reflects the importance of IFN-γ signaling in further differentiation of T cells into Th1 cells. We found that while the number of Th1 WT NR1 T cells and the number of Th1 IFN-γR^−/−^ NR1 T cells were comparable, there were substantially more Th1 IFN-γ^−/−^ NR1 T cells in the draining lymph node (Fig. 4A and B). Correlating with this, there were significantly higher numbers of activated Th1 IFN-γ^−/−^ NR1 T cells in the draining lymph node (Fig. 4C). However, the levels of activation of the Th1 IFN-γ^−/−^ NR1 T cells and Th1 WT NR1 T cells were similar (Fig. S3A), suggesting that the higher number of Th1 IFN-γ^−/−^ NR1 T cells was not due to enhanced activation. The levels of activated NR1 T cells also correlated with the number of activated endogenous CD4^+^ T cells (Fig. 4D), but there were no differences in the total numbers of endogenous CD4^+^ or total endogenous CD8^+^ T cells in the lymph nodes of these mice (Fig. S3B and C).

10.1128/mBio.00191-20.2 FIG S2 WT and IFN-γ^−/−^ NR1 T cells skew to a T-bet^+^ phenotype equally. (A and B) WT, IFN-γ^−/−^, and IFN-γR^−/−^ NR1 T cells were skewed *in vitro* to a Th1 phenotype. T-bet expression was analyzed using flow cytometry. Data are representative of results of two independent experiments performed with three replicates each and were analyzed using an ordinary one-way ANOVA with Dunnett’s multiple-comparison test. **, *P < *0.01. Download FIG S2, PDF file, 0.1 MB. Copyright © 2020 Helble et al. 2020 Helble et al. This content is distributed under the terms of the Creative Commons Attribution 4.0 International license.

10.1128/mBio.00191-20.3 FIG S3 Endogenous T cell populations in IFN-γ^−/−^ mice following NR1 T cell transfer. IFN-γ^−/−^ mice received 10^6^ Th1-skewed WT, IFN-γ^−/−^, or IFN-γR^−/−^ RFP NR1 T cells 1 day prior to infection with 5 × 10^6^ IFU C. trachomatis. (A to C) Five days postinfection, uterine draining lymph nodes were assessed by flow cytometry for (A) percent activated NR1 T cells (CD44^+^ CD62L^−^), (B) endogenous CD4^+^ T cells, and (C) endogenous CD8^+^ T cells. (D) Upper genital tracts were assessed by flow cytometry for endogenous CD8^+^ T cells. Data were pooled from results of two independent experiments performed with five mice per group and were analyzed using ordinary one-way ANOVA with Dunnett’s multiple-comparison test. Download FIG S3, PDF file, 0.2 MB. Copyright © 2020 Helble et al. 2020 Helble et al. This content is distributed under the terms of the Creative Commons Attribution 4.0 International license.

In the genital tract, we also observed higher levels of Th1 IFN-γ^−/−^ NR1 T cells accumulated in the tissue than of Th1 WT and Th1 IFN-γR^−/−^ NR1 T cells (Fig. 4E and F), which again correlated with the total number of endogenous CD4^+^ T cells (Fig. 4G) but not with the total number of endogenous CD8^+^ T cells (Fig. S3D). The mice that had received Th1 IFN-γ^−/−^ NR1 T cells actually had higher bacterial burden than the mice that received Th1 WT and Th1 IFN-γR^−/−^ NR1 T cells, with the latter showing protection from infection (Fig. 4H). Given these data, we believe that NR1 T cell production of IFN-γ (which can occur in Th1 WT and Th1 IFN-γR^−/−^ NR1 T cells) is critical to C. trachomatis clearance in mice without other sources of IFN-γ. In contrast, the mice that received Th1 IFN-γ^−/−^ NR1 T cells had higher bacterial burden, potentially leading to further recruitment and accumulation of NR1 and endogenous CD4^+^ T cells in the genital tract.

### Host IFN-γ production mediates bacterial clearance in the absence of IFN-γ production by NR1 T cells.

**FIG 5:**
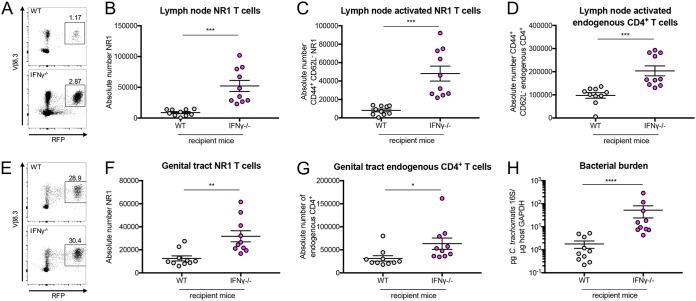
Host IFN-γ production mediates bacterial clearance in the absence of IFN-γ production by NR1 T cells. WT B6 or IFN-γ^−/−^ mice received 10^6^ Th1-skewed IFN-γ^−/−^ RFP NR1 T cells 1 day prior to infection with 5 × 10^6^ IFU C. trachomatis. (A to D) Five days postinfection, uterine draining lymph nodes were assessed by flow cytometry for (A and B) NR1 T cells, (C) activated NR1 T cells, and (D) activated endogenous CD4^+^ T cells. (E to H) Upper genital tracts were assessed by flow cytometry for (E and F) NR1 T cells and (G) endogenous CD4^+^ T cells and by qPCR for (H) bacterial burden. Data were pooled from results of two independent experiments performed with five mice per group and were analyzed using unpaired *t* test (B to D, F, and G) or Mann-Whitney test (H). *, *P < *0.05; **, *P < *0.01; ***, *P < *0.001; ****, *P < *0.0001.

**FIG 6:**
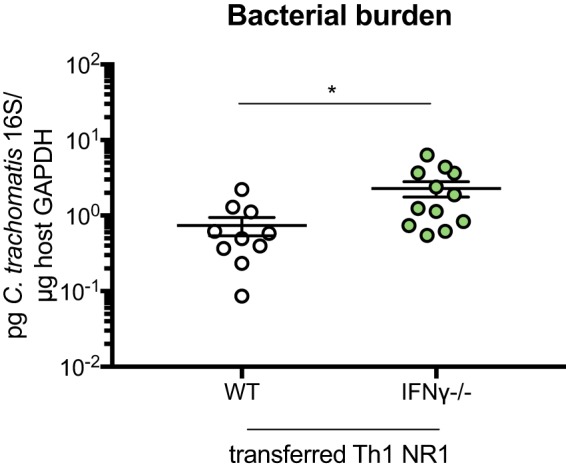
Host IFN-γ can synergize with IFN-γ produced by NR1 T cells to mediate enhanced bacterial clearance. WT B6 mice received 10^6^ Th1-skewed WT or IFN-γ^−/−^ RFP NR1 T cells 1 day prior to infection with 5 × 10^6^ IFU C. trachomatis. Five days postinfection, upper genital tracts were harvested and bacterial burden was assessed by qPCR. Data were pooled from results of two independent experiments performed with at least five mice per group and were analyzed using unpaired *t* test. *, *P < *0.05.

Given that NR1 T cell production of IFN-γ is critical for bacterial clearance, we next wondered if host production of IFN-γ would be similarly important. To test this, we transferred Th1-skewed IFN-γ^−/−^ NR1 T cells into either WT or IFN-γ^−/−^ mice prior to inoculation. At 5 days postinoculation, we found that the IFN-γ^−/−^ mice had substantially higher numbers of total Th1 IFN-γ^−/−^ NR1 T cells (Fig. 5A and B), activated Th1 IFN-γ^−/−^ T cells (Fig. 5C), total endogenous CD4^+^ and CD8^+^ T cells (Fig. S4A and B), and activated endogenous CD4^+^ T cells (Fig. 5D) in the draining lymph node than the WT B6 recipients. Similarly, there were significantly more Th1 IFN-γ^−/−^ NR1 T cells (Fig. 5E and F) and endogenous CD4^+^ T cells (Fig. 5G), but not endogenous CD8^+^ T cells (Fig. S4C), in the genital tract of IFN-γ^−/−^ mice and these mice also had higher bacterial burdens than WT B6 mice (Fig. 5H). Our data suggest that host IFN-γ production can help clear C. trachomatis in the absence of IFN-γ production from antigen-specific NR1 T cells. We also tested whether the effect of IFN-γ production by the host and the NR1 T cells could be synergistic by assessing bacterial burden in WT B6 mice that received Th1 WT NR1 T cells versus the burden in WT B6 mice that received Th1 IFN-γ^−/−^ NR1 T cells. Indeed, we found that the WT B6 mice with Th1 WT NR1 T cells had a significantly lower burden than the mice that received Th1 IFN-γ^−/−^ NR1 T cells (Fig. 6), suggesting that these dual sources of IFN-γ can be additive in aiding with bacterial clearance. Together, our data suggest that both endogenous CD4^+^ T cell IFN-γ production and antigen-specific production are important for clearing C. trachomatis.

10.1128/mBio.00191-20.4 FIG S4 Host IFN-γ production results in a decreased endogenous T cell response in the absence of IFN-γ production by NR1 T cells. WT or IFN-γ^−/−^ mice received 10^6^ Th1-skewed IFN-γ^−/−^ RFP NR1 T cells 1 day prior to infection with 5 × 10^6^ IFU C. trachomatis. (A and B) Five days postinfection, uterine draining lymph nodes were assessed by flow cytometry for (A) endogenous CD4^+^ T cells and (B) endogenous CD8^+^ T cells. (C) Upper genital tracts were assessed by flow cytometry for endogenous CD8^+^ T cells. Data were pooled from results of two independent experiments performed with at least five mice per group and were analyzed using unpaired *t* test. **, *P < *0.01; ***, *P < *0.001. Download FIG S4, PDF file, 0.1 MB. Copyright © 2020 Helble et al. 2020 Helble et al. This content is distributed under the terms of the Creative Commons Attribution 4.0 International license.

## DISCUSSION

IFN-γ is a critical cytokine for controlling *Chlamydia* infection. In this study, we first sought to address whether or not IFN-γ production or IFN-γ sensing was necessary for antigen-specific CD4^+^ T cells to C. trachomatis (NR1 T cells) to home to the genital tract. We found that NR1 T cells deficient in IFN-γ production or sensing had no defect in trafficking to the draining lymph node or genital tract following C. trachomatis infection (Fig. 1). It is possible that IFN-γ does not have an effect on naive NR1 T cell trafficking because these T cells use specific chemokine receptors and host integrins to traffic to the genital tract that are independent of IFN-γ (9, 10).

When we looked via microscopy, we found that there were segments of the genital tract that had different densities of NR1 T cells and that this segmentation was observed whether the NR1 T cells were from a WT, IFN-γ^−/−^, or IFN-γR^−/−^ background (Fig. 2). Not only did these segments containing higher numbers of NR1 T cells correlate with higher numbers of endogenous CD4^+^ and CD8^+^ T cells (Fig. 3), but the segments containing more NR1 T cells within the uterine horns also correlated with higher bacterial burden. Our data suggest not only that NR1 T cells can home to specific sites within the genital tract that contain C. trachomatis (which happens in an IFN-γ-independent manner) but also that there are specific segments in the genital tract that contain bacteria following transcervical inoculation. Whether or not this is reminiscent of human infection is unclear, but it does suggest that in the transcervical mouse model of C. trachomatis inoculation, there are localized inflammatory pockets within the genital tract that contain both host T cells and antigen-specific CD4^+^ T cells.

Other groups have also used microscopy to visualize antigen-specific CD8^+^ T cell responses to lymphocytic choriomeningitis virus (LCMV) in the female reproductive tract of mice (20). Those studies showed that antigen-specific CD8^+^ T cells do localize in sections of the genital tract containing LCMV, correlating with our findings with C. trachomatis. Given this, it is possible that transgenic CD8^+^ T cells specific for C. trachomatis protein CrpA (7) or transgenic CD4^+^ T cells corresponding to C. muridarum (8) would similarly be able to home to the specific sites within the genital tract that contain bacteria. Future work will address whether or not this is an NR1 or transcervical inoculation-specific phenomenon within the context of *Chlamydia* infection. Indeed, transcervical inoculation and intravaginal inoculation with C. muridarum elicit different immune responses in the upper genital tract (21), and future work will determine if the inflammatory sections in the upper genital tract that occur with C. trachomatis transcervical inoculation also occur with C. muridarum intravaginal inoculation following ascension.

We also used IFN-γ^−/−^ NR1 T cells to definitively show that IFN-γ production by antigen-specific Th1 T cells is sufficient to control C. trachomatis infection (Fig. 4). While this has previously been shown indirectly (8, 14, 15, 22), our use of IFN-γ^−/−^ NR1 T cells confirms the importance of IFN-γ production during C. trachomatis infection. Interestingly, IFN-γR^−/−^ NR1 T cells were still able to protect mice, suggesting that their ability to produce IFN-γ was enough to help clear infection. This was the case despite the fact that these T cells were unable to be skewed to a T-bet^+^ phenotype as effectively as WT NR1 T cells, suggesting that the cells in the subset were T-bet^+^ were perhaps able to produce sufficient levels of IFN-γ to mediate protection. It is known that IFN-γ sensing can play an important role in shaping the Th1 response, as it can act in an autocrine manner with respect to T cells by allowing them to further differentiate into Th1 T cells (23). Indeed, Th1 IFN-γR^−/−^ NR1 T cells showed slightly lower levels of activation and of T-bet^+^ phenotype than WT Th1 NR1 T cells in the draining lymph node (see Fig. S2 and S3A in the supplemental material). However, since the ability to sense IFN-γ was not critical for bacterial clearance, it is possible that because IFN-γR^−/−^ T cells were previously skewed to a Th1 phenotype *in vitro* prior to transfer, this feedforward loop of IFN-γ signaling was not necessary for the T cells to be protective and that the protection was solely driven by the production of IFN-γ by these T cells. Mice that received IFN-γ^−/−^ NR1 T cells showed an accumulation of both endogenous CD4^+^ T cells and IFN-γ^−/−^ NR1 T cells in the genital tract and, to a lesser extent, draining lymph nodes. We suspect that the reason for NR1 T cell accumulation is the antigen-specific nature of these cells and that this process occurs cyclically. Specifically, IFN-γ^−/−^ NR1 T cells (and endogenous CD4^+^ T cells that are likely antigen specific) become activated through the activity of C. trachomatis antigen and travel to the genital tract, where they are unable to clear infection because of their inability to produce IFN-γ. As a result, bacterial burden and antigen levels remain high and more IFN-γ^−/−^ NR1 T cells are activated by C. trachomatis antigen, leading to an accumulation of these cells in the tissue and a lack of bacterial clearance (Fig. S5). While it is possible that the endogenous CD4^+^ T cells that accumulate in the lymph nodes and genital tract are specific to C. trachomatis, these T cells could also be specific to non-C. trachomatis antigen and accumulate in the tissues simply as a product of inflammation caused by infection. Determining the identity of these endogenous CD4^+^ T cells, as well as the inflammatory signals that lead to their accumulation in the tissues, can help to further elucidate their role during C. trachomatis infection. The lack of a difference in the levels of endogenous CD8^+^ T cell accumulation is consistent with the fact that CD8^+^ T cells are not naturally protective against C. trachomatis genital infection in mice (24, 25).

10.1128/mBio.00191-20.5 FIG S5 Experimental model. Download FIG S5, PDF file, 0.04 MB. Copyright © 2020 Helble et al. 2020 Helble et al. This content is distributed under the terms of the Creative Commons Attribution 4.0 International license.

While IFN-γ production by antigen-specific T cells is important for clearing C. trachomatis infection, we also found that in the absence of IFN-γ production by NR1 T cells, host IFN-γ in WT mice can reduce bacterial burden. We speculate that this IFN-γ production is driven by endogenous CD4^+^ T cells, as had been previously shown to be the case following pulmonary C. muridarum infection in neonatal mice (26) and intravaginal infection with C. muridarum (17, 24, 27). While either endogenous CD4^+^ T cell production of IFN-γ or NR1 T cell production of IFN-γ can be beneficial with respect to bacterial clearance compared to a complete absence of IFN-γ production, having IFN-γ production by both NR1 T cells and endogenous T cells offers a slight advantage compared to production by one or the other (Fig. 6). While this effect is significant, it is unsurprising that it is modest. Given that antigen-specific CD4^+^ T cells and endogenous cells produce sufficient IFN-γ individually to mediate bacterial clearance, the additional IFN-γ production that occurs when the two sources are combined can enhance clearance to only a limited degree. Our data further confirm that IFN-γ is a critical cytokine that is required for bacterial clearance.

Ultimately, understanding the importance of IFN-γ production and sensing by antigen-specific T cells is essential to developing an effective vaccine against C. trachomatis. Here, we show that IFN-γ production and sensing are not required for the homing of antigen-specific T cells to the sites of infection. Using two-photon microscopy, we found that antigen-specific T cell receptors are critical for sensing C. trachomatis antigen in specific pockets in the upper genital tract. Our work provides valuable insight into the mouse model of infection and emphasizes how critical CD4^+^ T cells are for clearance, and further work is necessary to determine whether or not this translates to human infection.

## MATERIALS AND METHODS

### Growth and isolation of bacteria.

Chlamydia trachomatis serovar L2 (434/Bu; ATCC) was propagated in McCoy cells as described previously (9, 28). Aliquots of purified elementary bodies were stored at –80°C in SPG buffer (250 mM sucrose, 10 mM sodium phosphate, 5 mM l-glutamic acid) and thawed immediately prior to use.

### Mice.

Six-to-8-week-old C57BL/6J mice, B6.129S7-*Ifng^tm1Ts^*/J mice (IFN-γ^−/−^), B6.129P2(Cg)-*Cdh1^tmCle^*/J mice (E-cadherin–monomeric cyan fluorescent protein [E-cadherin–mCFP]), and B6.Cg-Tg(CAG-mRFP1)1F1Hadj/J mice (β-actin RFP) were purchased from Jackson Laboratory (Bar Harbor, ME). NR1 mice that recognize the peptide from the C. trachomatis antigen Cta1 (residues 133 to 152) have been previously described (6). To generate RFP NR1 mice, RFP and NR1 mice were crossed and assessed by flow cytometry for RFP expression. To generate RFP IFN-γ^−/−^NR1 mice or RFP IFN-γR^−/−^ NR1 mice, RFP NR1 mice were crossed with either IFN-γ^−/−^ or IFN-γR^−/−^ mice. IFN-γ^−/−^ or IFN-γR^−/−^ status was determined by PCR, while RFP expression determined by flow cytometry. All mice were housed in the Harvard Medical School Center for Animal Resources and Comparative Medicine, and all experiments were approved by Harvard’s Institutional Animal Care and Use Committee.

### T cell adoptive transfers.

For naive NR1 transfers, CD4^+^ T cells were isolated from secondary lymphoid organs from RFP WT NR1 mice, RFP IFN-γ^−/−^ NR1 mice, or RFP IFN-γR^−/−^ NR1 mice. For Th1-skewed NR1 T cells, CD4^+^ T cells were purified from secondary lymphoid organs of RFP WT NR1 mice, RFP IFN-γ^−/−^ NR1 mice, or RFP IFN-γR^−/−^ NR1 mice using a Dynabeads Untouched mouse CD4 cell kit (Invitrogen). T cells were stimulated using irradiated splenocytes from a C57BL/6 mouse that had been pulsed with 5 μM Cta1 peptide (Cta1_133-152_) at a 4:1 stimulator/T cell ratio. Cells were cultured for 5 days in RPMI 1640 (Invitrogen) supplemented with 10% fetal calf serum, l-glutamine, HEPES, 50 mM 2-mercaptoethanol, 50 U/ml penicillin, and 50 mg/ml streptomycin along with 10 ng/ml interleukin-12 (IL-12; Peprotech, Rocky Hill, NJ) and 10 μg/ml anti-IL-4 (Bio X Cell, West Lebanon, NH). A total of 10^6^ total naive or Th1-skewed NR1 T cells were injected intravenously in a 200-μl volume into recipient mice 1 day prior to infection with C. trachomatis.

### Infection of mice and preparation of tissue.

At 1 week prior to infection, mice were treated subcutaneously with 2.5 mg medroxyprogesterone. Mice were infected transcervically with 5 × 10^6^ inclusion-forming units (IFUs) of C. trachomatis mixed in 10 μl SPG buffer or were inoculated with 10 μl phosphate-buffered saline (PBS) using an NSET pipette tip (ParaTechs, Lexington, KY). At specific time points postinfection, upper genital tracts (uterine horns and ovaries) and draining iliac lymph nodes were harvested. Brachial lymph nodes were used for nondraining lymph node experiments. Single-cell suspensions of lymph nodes were prepared by grinding the tissue between frosted microscope slides. Upper genital tracts were minced with scalpels, incubated in Hanks balanced salt solution (HBSS)/Ca2^+^/Mg2^+^ containing 1 mg/ml type XI collagenase and 50 Kunitz units/ml DNase for 30 min at 37°C and then washed in Ca2^+^/Mg2^+^-free PBS containing 5 mM EDTA. Tissues were then ground between frosted microscope slides prior to filtration through 70-μm-pore-size mesh. For experiments that required dividing the uterine horns into NR1^hi^ and NR1^lo^ sections, tissues were harvested and cut open using scissors to expose the lumen. T cell clusters were visualized using a Leica MZ10 F fluorescence imaging microscope (Leica Microsystems, Wetzlar, Germany) and were cut into NR1^hi^ (visible RFP T cells) and NR1^lo^ (no visible RFP T cells) segments accordingly. Tissue pieces were then enzymatically dissociated with HBSS/Ca2^+^/Mg2^+^ containing 0.5 mg/ml type XI collagenase and 25 Kunitz units/ml DNase and then ground between frosted microscope slides and filtered as described above.

### Flow cytometry.

All antibodies were purchased from BioLegend except where otherwise noted. Following isolation, cells were stained with fluorochrome-conjugated antibodies against mouse CD3 (clone 17A2), CD4 (clone GK1.5), CD8 (clone 53-6.7), CD44 (clone 1M7), CD62L (clone Mel-14), and T cell receptor Vβ8.3 (clone 1B3.3) (BD Biosciences) along with anti-FcRγ (Bio X Cell, West Lebanon, NH) and a LIVE/DEAD Fixable Aqua Dead cell stain kit to exclude dead cells (Invitrogen). To assess T-bet^+^ expression, a subset of skewed cells were fixed and permeabilized using an Invitrogen transcription factor fixation/permeabilization kit (Invitrogen) and staining with an anti-T-bet antibody (clone 4B10). AccuCheck counting beads (Invitrogen) were used to determine absolute cell counts. Data were collected on an LSR II flow cytometer (BD Biosciences) and were analyzed using FlowJo (Tree Star, Ashland, OR).

### Two-photon microscopy and image processing.

After upper genital tract harvest, the cervix and uterine horns were cut open using scissors to expose the lumen of the tissue. Tissues were placed into a tissue-embedding cassette and fixed in 4% paraformaldehyde (PFA) for at least 6 h at 4°C. Tissues were washed in PBS for at least 1 h at 4°C prior to mounting. Tissues were mounted (whole mount) for microscopy using GenTeal lubricant eye gel. Two-photon microscopy imaging of unsectioned fixed tissues was conducted using an upright microscope (Prairie Technologies) with a MaiTai Ti:sapphire laser (Spectra-Physics) tuned to 900 nm. Bandpass filters of 455/70 nm (CFP) and 590/50 nm (mRFP) were used to filter emitted light before detection. Z-stacks were obtained up to a depth of 100 μm and were processed to obtain maximum projections using Imaris 9.2.1 (Bitplane, Belfast, United Kingdom) (29).

### Quantitative PCR.

Bacterial burden was determined through quantitative PCR as previously described (30). Briefly, DNA was extracted from genital tract tissue samples using a QIAamp DNA minikit (Qiagen). *Chlamydia* 16S DNA and mouse GAPDH (glyceraldehyde-3-phosphate dehydrogenase) were quantified using primer pairs and dually labeled probes (IDT, San Jose, CA, or Applied Biosciences).

### Statistical analysis.

Statistical analysis was performed using Prism software (GraphPad). Differences were considered statistically significant if the *P* value was less than 0.05. All data are represented as means ± standard errors of the means (SEM).
