# Supplementary figures and images for: Gamma Interferon Is Required for Chlamydia Clearance but Is Dispensable for T Cell Homing to the Genital Tract
Source: mBio. 2020 Mar 17;11(2):e00191-20. doi: 10.1128/mBio.00191-20 (PMC7078466; doi:10.1128/mBio.00191-20)

**A**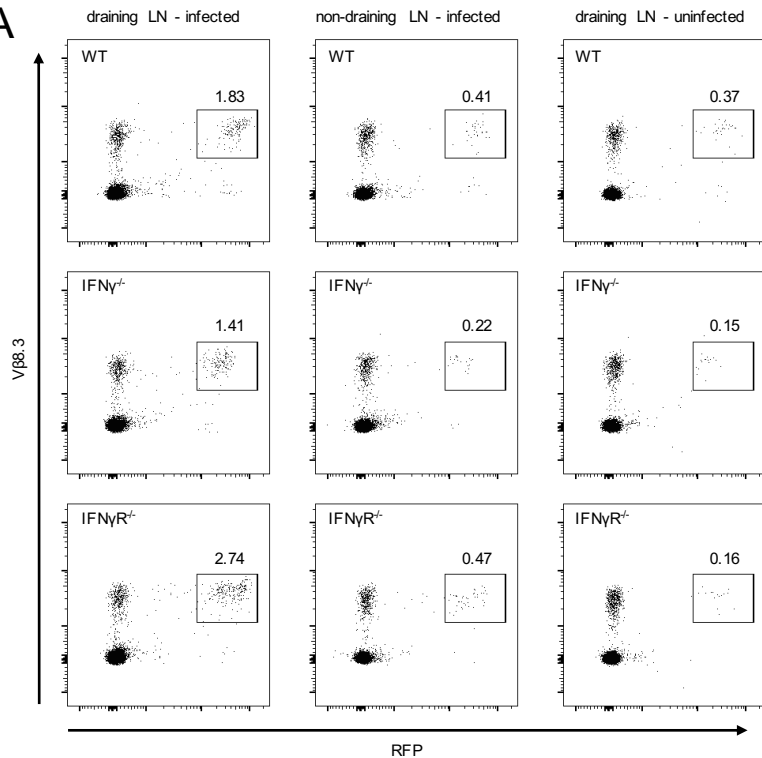**B**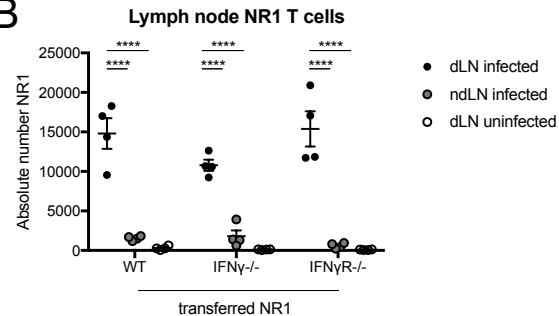**C**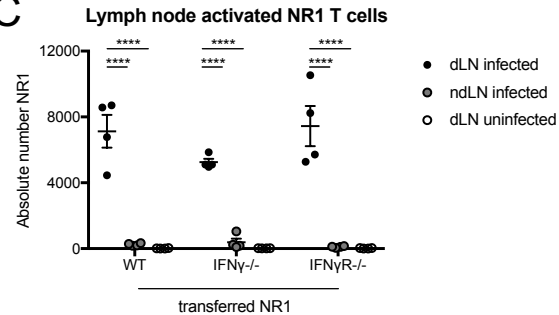

Supplement: FIG S1 [file mBio.00191-20-sf001.pdf]

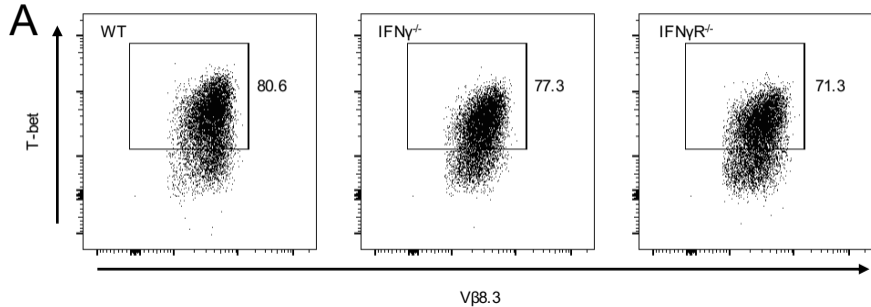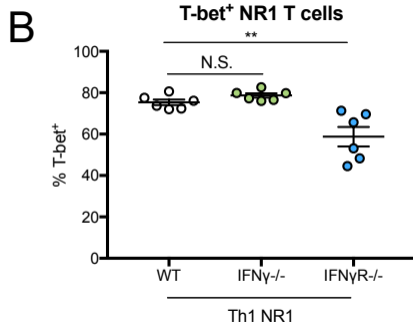

Supplement: FIG S2 [file mBio.00191-20-sf002.pdf]

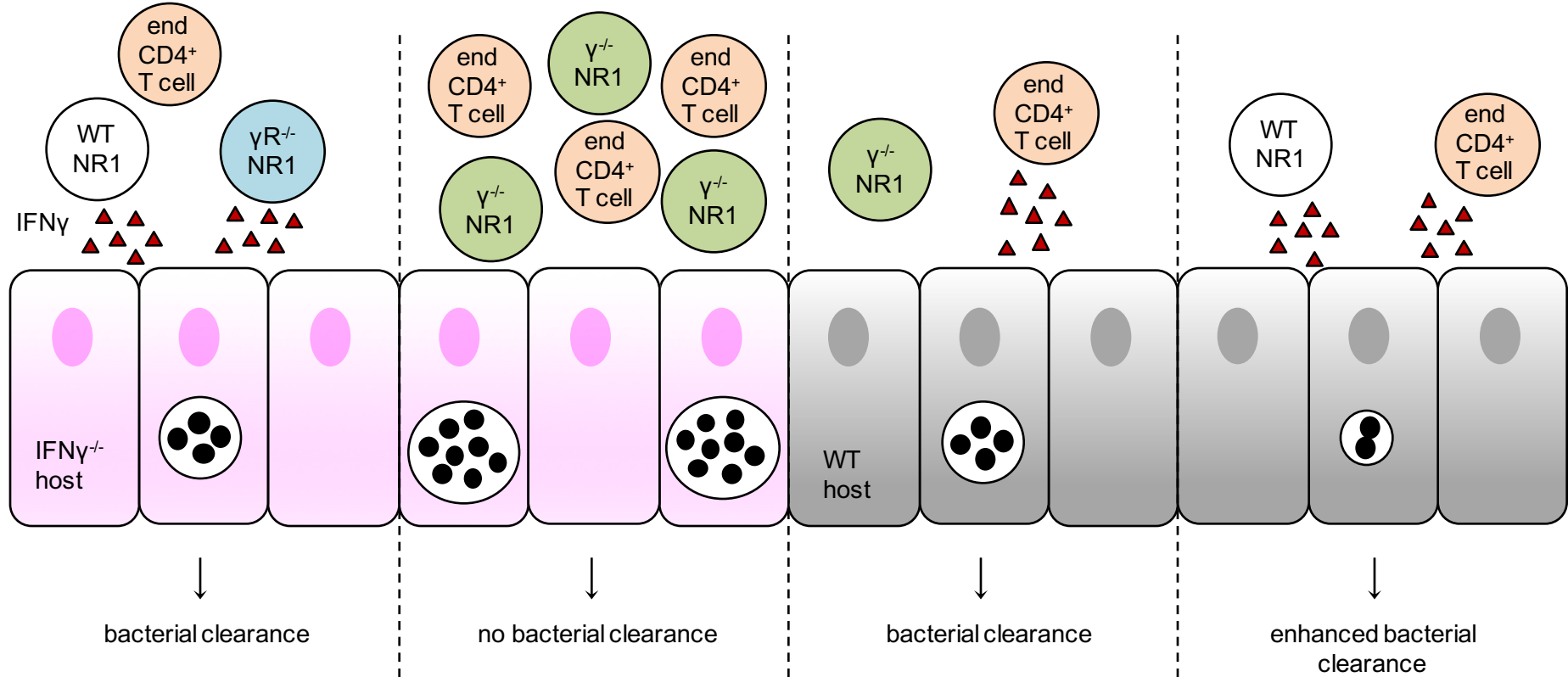

Supplement: FIG S5 [file mBio.00191-20-sf005.pdf]
