# Supplementary material for: Gamma Interferon Is Required for Chlamydia Clearance but Is Dispensable for T Cell Homing to the Genital Tract
Source: mBio. 2020 Mar 17;11(2):e00191-20. doi: 10.1128/mBio.00191-20 (PMC7078466; doi:10.1128/mBio.00191-20)
Supplement: FIG S3 [file mBio.00191-20-sf003.pdf]

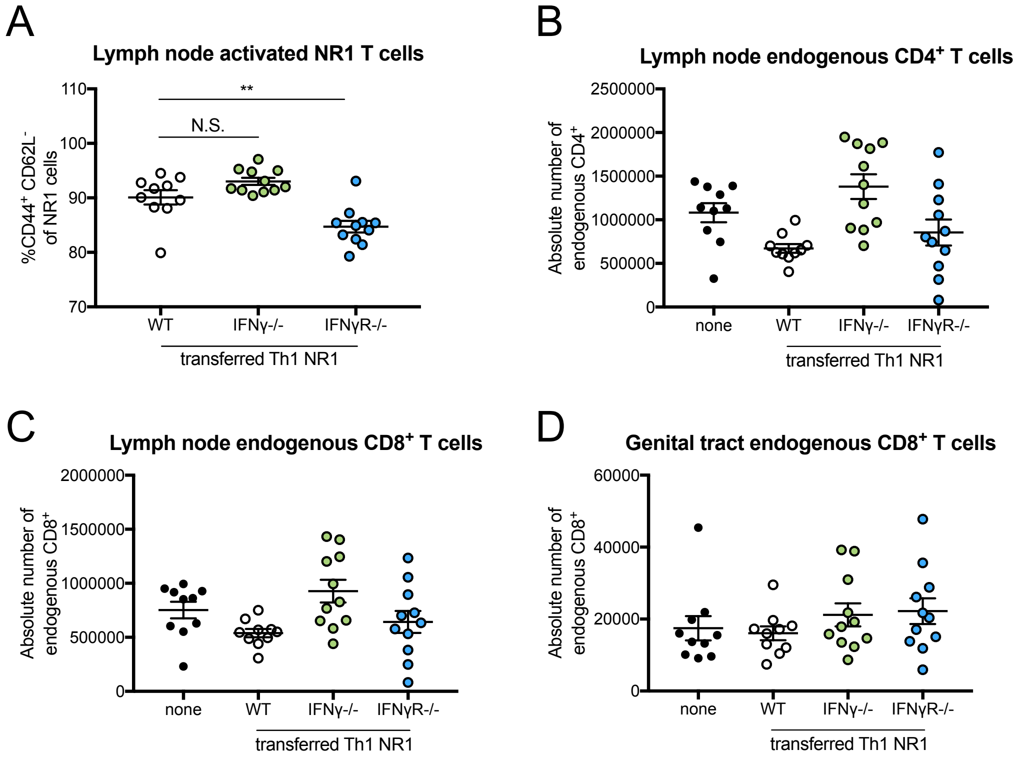
A
Lymph node activated NR1 T cells
%CD44+ CD62L- of NR1 cells
110
100
90
80
70
**
N.S.
WT
IFNγ-/-
IFNγR-/-
transferred Th1 NR1
B
Lymph node endogenous CD4+ T cells
Absolute number of endogenous CD4+
2500000
2000000
1500000
1000000
500000
0
none
WT
IFNγ-/-
IFNγR-/-
transferred Th1 NR1
C
Lymph node endogenous CD8+ T cells
Absolute number of endogenous CD8+
2000000
1500000
1000000
500000
0
none
WT
IFNγ-/-
IFNγR-/-
transferred Th1 NR1
D
Genital tract endogenous CD8+ T cells
Absolute number of endogenous CD8+
60000
40000
20000
0
none
WT
IFNγ-/-
IFNγR-/-
transferred Th1 NR1
