# Supplementary material for: Gamma Interferon Is Required for Chlamydia Clearance but Is Dispensable for T Cell Homing to the Genital Tract
Source: mBio. 2020 Mar 17;11(2):e00191-20. doi: 10.1128/mBio.00191-20 (PMC7078466; doi:10.1128/mBio.00191-20)
Supplement: FIG S4 [file mBio.00191-20-sf004.pdf]

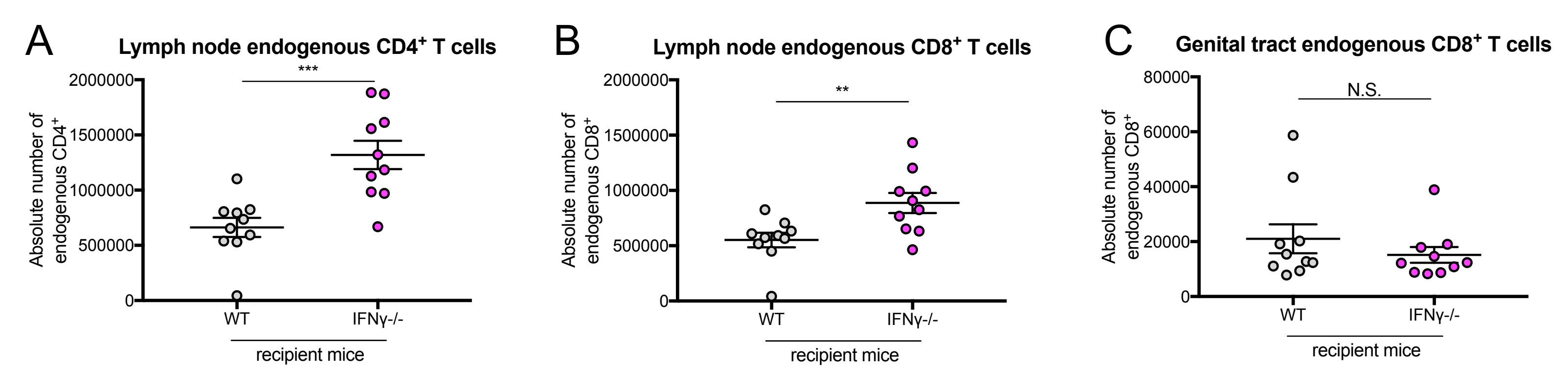
A
Lymph node endogenous CD4+ T cells
Absolute number of endogenous CD4+
2000000
1500000
1000000
500000
0
***
WT
IFNγ-/-
recipient mice
B
Lymph node endogenous CD8+ T cells
Absolute number of endogenous CD8+
2000000
1500000
1000000
500000
0
**
WT
IFNγ-/-
recipient mice
C
Genital tract endogenous CD8+ T cells
Absolute number of endogenous CD8+
80000
60000
40000
20000
0
N.S.
WT
IFNγ-/-
recipient mice
